# Allergic Airway-Induced Hypersensitivity Is Attenuated by Bergapten in Murine Models of Inflammation

**DOI:** 10.1155/2019/6097349

**Published:** 2019-02-03

**Authors:** Douglas B. Aidoo, David D. Obiri, Newman Osafo, Aaron O. Antwi, Leslie B. Essel, Babatunde M. Duduyemi, Martins Ekor

**Affiliations:** ^1^Department of Pharmacology, Faculty of Pharmacy and Pharmaceutical Sciences, College of Health Sciences, Kwame Nkrumah University of Science & Technology, KNUST, Kumasi, Ghana; ^2^Division of Pharmacology and Toxicology, School of Pharmacy, University of Missouri, Kansas City, USA; ^3^Department of Pathology, School of Medical Sciences, College of Health Sciences, Kwame Nkrumah University of Science & Technology, KNUST, Kumasi, Ghana; ^4^Department of Pharmacology, School of Medical Sciences, College of Health & Allied Sciences, University of Cape Coast, Cape Coast, Ghana

## Abstract

Bergapten (5-methoxypsoralen, 5-MOP) is a plant-derived furocoumarin with demonstrated anti-inflammatory action. The present study investigated its effects on allergic inflammation in two related pathways of mast cell degranulation. Compound 48/80 and lipopolysaccharide (LPS) were used to activate the IgE-independent pathway while bovine serum albumin (BSA) was used as allergen for the IgE-dependent pathway. The modulatory effect of bergapten on mast cell degranulation, neutrophil extravasation, protein concentration, lung histopathology, and oxidative stress was assessed. Bergapten at 10, 30, and 100 *μ*g/ml for 15 min stabilized mast cells in rat mesenteric tissue from disruption *in vitro* and when administered *in vivo* at 3, 10, and 30 mg kg^−1^ for 1 h protected mice from fatal anaphylaxis induced by compound 48/80. Similarly, treatment of LPS-challenged mice with bergapten (3, 10, and 30 mg kg^−1^) for 24 h significantly decreased neutrophil infiltration into bronchoalveolar lavage fluid, mean protein concentration, and inflammatory cell infiltration of pulmonary tissues when compared to the saline-treated LPS-challenged control. In addition, lung histology of the bergapten-treated LPS-challenged mice showed significantly less oedema, congestion, and alveolar septa thickening when compared to the saline-treated LPS-challenged disease control. LPS-induced oxidative stress was significantly reduced through increased tissue activities of catalase and superoxide dismutase and reduced malondialdehyde levels on treatment with bergapten. In the triple antigen-induced active anaphylaxis, daily administration of bergapten at 3, 10, and 30 mg kg^−1^ for 10 days, respectively, protected previously sensitized and challenged mice against anaphylactic shock. Overall, our study demonstrates the ability of bergapten to attenuate allergic airway-induced hypersensitivity in murine models of inflammation, suggesting its possible therapeutic benefit in this condition.

## 1. Introduction

Allergy is one of the common diseases which affect mankind, and it is also responsible for significant morbidity and mortality. The prevalence of allergic diseases such as anaphylaxis, asthma, rhinitis, and atopic dermatitis has increased in recent times despite the general health improvement in the population [1, 2].

Anaphylaxis, a Type I hypersensitivity reaction is an acute allergic response triggered by the release of chemical mediators from mast cells and basophils on activation [3]. A pathway of mast cell activation described as the peptidergic pathway is not IgE-dependent, and it is activated by basic secretagogues which are polycationic compounds and include compound 48/80 [4]. Among the several surface receptors expressed on the mast cell is the receptor with high affinity for the antibody immunoglobulin E (IgE). This receptor, also known as the Fc epsilon RI (FcεRI), mediates the IgE pathway of mast cell activation [5, 6]. Stimulation of mast cells by either the IgE-independent or IgE-dependent mechanisms triggers the activation of signal transduction pathways which initiates a cascade of biochemical events, leading to the rapid release of inflammatory mediators. These include histamine, proteases, eicosanoids, and cytokines such as tumor necrosis factor alpha (TNF_*α*_) and interleukins (IL-6, IL-8, IL-4, and IL-13), respectively. Together, these preformed and *de novo* synthesized mediators cause allergic inflammation and anaphylactic reactions [7].

The available treatment options for allergic diseases presently include the use of inhaled corticosteroids such as fluticasone, mast cell stabilizers examples of which are nedocromil, leukotriene inhibitors examples of which are zileuton and zafirlukast, and the long-acting *β*_2_-adrenergic agonists such as albuterol. Unfortunately, the limitations to the therapeutic success in managing these allergic conditions are largely associated with the adverse effects of the drugs [8–12], thus making the search for other regimens with less or no side effects imperative. Alternatives from natural sources have become the current focus in research. One of such plant-derived product is bergapten (5-methoxypsoralen, 5-MOP), a furocoumarin, used by the cosmetic and pharmaceutical industries to treat dermatological conditions such as psoriasis and vitiligo [13, 14]. Evidence from both experimental and clinical investigations have reported on its anti-inflammatory [15, 16], antiproliferative [17], and anticancer effects [18, 19]. Informed by its already established anti-inflammatory effects, we sought to investigate the potential benefit of bergapten in the treatment of allergic hypersensitivity reactions in murine models of inflammation.

## 2. Materials

### 2.1. Animals

Male C57BL/6 mice (25–30 g) and Sprague Dawley rats (250–300 g) were purchased from the Noguchi Memorial Institute for Medical Research, University of Ghana, Legon, and housed under standard laboratory conditions (temperature 25 ± 2°C with a 12 h light-dark cycle) in the animal house of the Department of Pharmacology, Faculty of Pharmacy and Pharmaceutical Sciences, KNUST, Ghana. Experimental animals were handled in compliance with the Animal Welfare Regulations (USDA 1985; US Code, 42 USC *§* 289d) and the Public Health Service Policy on Humane Care and Use of Laboratory Animals (PHS 2002). Use of the experimental animals was approved by the Ethical Review Committee of the Faculty of Pharmacy and Pharmaceutical Sciences, KNUST.

### 2.2. Drugs and Chemicals

Bergapten (5-methoxypsolaren, 5-MOP), compound 48/80 (C2313), lipopolysaccharide (LPS); *Escherichia coli*, serotype (O55:B5), and dexamethasone were procured from Sigma-Aldrich (St Louis, USA). Bovine serum albumin (BSA) was purchased from PAA Laboratories (Marburg, Germany), Diphtheria Pertussis Tetanus (DPT) vaccine was obtained from the Immunization Unit, South Suntreso Government Hospital (Kumasi, Ghana), trichloroacetic acid (TCA), thiobarbituric acid (TBA), potassium dichromate, sodium bicarbonate, sodium dihydrogen orthophosphate monohydrate, chloroform, and disodium hydrogen phosphate were procured from BDH Chemicals (England, UK) Complete Protease Inhibitor Cocktail Tablet was purchased from Santa Cruz Biotechnology (Dallas, TX, USA).

## 3. Methods

### 3.1. Compound 48/80-Induced Mast Cell Degranulation in Rat Mesenteric Tissue *In Vitro*

The method of Kaley and Weiner [20] was followed with slight modifications. A Sprague Dawley rat was sacrificed and pieces of its mesentery tissue excised and collected into 6 different Petri dishes each containing 10 ml Ringer–Locke solution. Into the respective Petri dishes (I–VI), 100 *µ*l of the following was added:

  I: Ringer–Locke
  II: normal saline (0.9% w/v NaCl)
  III: disodium cromoglycate (10 *μ*g ml^−1^)
  IV–VI: bergapten 10, 30, and 100 *μ*g ml^−1^, respectively

Samples were incubated for 15 min at 37°C. Test tissues with the exception of the Ringer–Locke-treated naïve control were further incubated for 10 min with 1 *µ*g C48/80. All tissues were immersed in formaldehyde solution (4%) containing toluidine blue (0.1%, pH 2.5) for 30 min and transferred through acetone and xylene twice for fixation and staining of mast cells, respectively. Tissues were mounted on slides and observed under a high power light microscope (Leica DM2500 M) at ×40 magnification. Mast cells were counted and the percentage protection from degranulation was calculated in five randomly selected fields.

### 3.2. Compound 48/80-Induced Systemic Anaphylaxis in Mice

Systemic anaphylaxis was induced following the method earlier described by Choi et al. [21] with slight modifications. C57 BL/6 mice (25–30 g) were randomized into 5 groups (*n* = 5) and given one of the following treatments:

  Group I (naïve control): normal saline 10 ml kg^−1^, *p.o.*
  Group II (positive control): disodium cromoglycate 50 mg kg^−1^, *i.p.*
  Groups III–V (test groups): bergapten 3, 10 and 30 mg kg^−1^, *p.o.*, respectively

Mice received C48/80 (8 mg kg^−1^, *i.p*.) 1 h later and monitored for death due to anaphylactic shock within 1 h. Total number of deaths in each group was expressed as percentage mortality using the following formula:

(1) $$\begin{array}{c} \%\text{ mortality}=\left[\frac{\text{number of dead mice}}{\text{total number of mice in the group}}\right]\times 100. \end{array}$$

### 3.3. Lipopolysaccharide-Induced Lung Inflammation

The modified method of Lowry [22] was followed. C57BL/6 mice (25–30 g) were randomly placed into 6 groups (*n* = 5) and given one of the following treatments:

  Group I (naïve control): normal saline 10 ml kg^−1^, *i.p.*
  Group II (disease control): normal saline 10 ml kg^−1^, *i.p*
  Group III (positive control): dexamethasone 10 mg kg^−1^, *i.p.*
  Groups IV–VI: bergapten 3, 10, and 30 mg kg^−1^, *p.o.*, respectively

Test mice were challenged with LPS (*Escherichia coli*, O55:B5, 0.5 mg ml^−1^) via aerosol for 30 min while saline-treated naïve control mice received PBS (10 ml kg^−1^, *i.p.*) only. Mice were sacrificed 24 h later by cervical dislocation.

#### 3.3.1. Bronchoalveolar Lavage Fluid (BALF) Collection and Analysis

The trachea was carefully exposed through a midline incision, and the lungs were washed 3× with 1 ml of cold normal saline avoiding contamination of luminal contents with blood and damage to the lung tissues. The trachea was cannulated, and bronchoalveolar lavage fluid (BALF) was collected by aspiration with a Pasteur pipette into Eppendorf tubes and used for neutrophil count, and protein content estimation is as follows:

*(1) Neutrophil Count*. Neutrophil count in the recovered BALF was carried out in triplicates on each sample using an automated analyser (Sysmex KX-21N, Sysmex America Inc., Illinois, USA).

*(2) Total Protein Content*. Bronchoalveolar lavage fluid was centrifuged (Wagtech, C257-120, UK) at 258 × *g* for 10 min at 4°C. The cell-free supernatant was used for the determination of total protein concentration in triplicates using an automated Clinical analyser (Flexor Junior, Vital Scientific B.V., Netherlands).

#### 3.3.2. Histology of Lung Tissue and Pulmonary Cell Infiltration

The left lobe of each lung was carefully removed and immediately fixed in 10% buffered formalin. Lung tissues were processed in an automatic tissue processor (TP 1020, Leica Biosystems, Wetzlar, Germany), serially dehydrated in ethanol, cleared in xylene, and embedded in paraffin using a Leica EG 1160 embedding machine (Leica Biosystems, Wetzlar, Germany). Transverse sections of 3 *µ*m was made with a microtome (RM 2155 RTS, Leica Biosystems, Wetzlar, Germany). The tissues were stained with haematoxylin and eosin (H&E), mounted on slides and viewed under a digital light microscope (DM 750, Leica Microsystems, Wetzlar, Germany) fitted with a digital camera (ICC 50 HD, Leica Microsystems, Wetzlar, Germany). Histopathological findings were observed in a blinded manner. Quantitative analysis was performed with ImageJ analysis tool (version 1.50i). A method described by Zare et al. [23] with modification was used to assess the degree of pulmonary cell infiltration. The scoring system was as follows: 0, no cell; 1, few cells; 2, a ring of cells 1 cell layer deep; 3, a ring of cells, 2–4 cell layers deep; and 4, a ring of cells >4 cell layers deep in the peribronchiolar and perivascular regions. Alveolar cell infiltration was assessed as follows: 0, no cell infiltrates or widen septa; 1, few infiltrates with widen septa; 2, cell infiltrates with widening septa; and 3, filled alveolar air spaces with thickened septa. Scores for peribronchiolar, perivascular, and alveolar cell infiltration were summed into 11–point composite score.

### 3.4. Assay for Oxidative Stress Markers

Systemic inflammation was induced with LPS in C57BL/6 mice employing the modified method of Lowry [22] as described earlier. The right lobe of each lung was washed with PBS and quickly stored at −80°C until used. The frozen lungs were thawed and homogenized in ice-cold buffer (Triton X-100 (1%), protease inhibitor cocktail, Tris HCl (150 mM), NaCl (150 mM), and glycerol (10%), pH 7.4) to obtain a 10% w/v homogenate. The homogenate was centrifuged (Wagtech, C257-120, UK) for 20 min at 6440 × *g*. The amount of protein in the supernatant was quantified using the Bradford method. Supernatant was carefully pipetted into clean Eppendorf tubes and stored at −80°C. In triplicate, aliquot of the supernatant was subjected to biochemical assays using Synergy H1 Hybrid Multi-Mode Microplate Reader (BioTek Technologies, Winooski, VT, USA) for the following oxidative stress markers.

#### 3.4.1. Catalase (CAT)

The method of Sinha [24] with slight modification was used to determine catalase activity. The principle was based on the ability of catalase to hydrolyze hydrogen peroxide (H_2_O_2_) to water (H_2_O) and molecular oxygen (O_2_). Briefly, to a 100 *µ*l aliquot of tissue supernatant, 1 ml phosphate buffer (0.01 M, pH 7.0) and 400 *µ*l H_2_O_2_ (1.18 M) were added. The mixture was incubated at room temperature for 5 min. 2 ml mixture (3:1) of glacial acetic acid and dichromate (5%) was added and absorbance measured at 620 nm. One unit of catalase activity, defined as the amount of enzyme that degrades 1 mmol H_2_O_2_ per min at 25°C and pH 7.0, was expressed in terms of its molar extinction coefficient, 39.4 M^−1^ cm^−1^.

(2) $$\begin{array}{c} \frac{\text{mUnit of CAT}}{\text{mg protein}}=\left[\frac{\text{absorbance }620\text{ nm}}{3.94\times \text{weight of protein}}\right]\times 1000. \end{array}$$

#### 3.4.2. Superoxide Dismutase (SOD)

Activity of superoxide dismutase was determined following the method described by Misra and Fridovich [25], with slight modifications. Briefly, 500 *µ*l tissue supernatant was added to 150 *µ*l ice-cold chloroform and 750 *µ*l ethanol (96% v/v), vortexed for 1 min, and then centrifuged (Wagtech, C257-120, UK) at 716 × *g* for 20 min. To 500 *µ*l of the resulting supernatant, 500 *µ*l EDTA (0.6 mM) and 1 ml carbonated bicarbonate buffer (0.1 M, pH 10.2) were added. The reaction was initiated by the addition of 50 *µ*l adrenaline (1.3 mM). Absorbance was measured at 480 nm against a blank. Activity of SOD, measured as the quantity of the enzyme required to inhibit the auto-oxidation of adrenaline, was calculated using the following equation:

(3) $$\begin{array}{c} \%\text{ inhibition}=\left[\frac{\text{absorbance}\left(\text{test}\right)-\text{absorbance}\left(\text{blank}\right)}{\text{absorbance}\left(\text{test}\right)}\right]\times 100. \end{array}$$

SOD activity was expressed in units per mg protein, where 1 unit of enzyme activity is the quantity of enzyme required to prevent the auto-oxidation of adrenaline at 25°C calculated with the following equation:

(4) $$\begin{array}{c} \frac{\text{units of SOD activity}}{\text{mg protein}}=\left[\frac{\%\text{ inhibition}}{50\times \text{weight of protein}}\right]. \end{array}$$

#### 3.4.3. Lipid Peroxidation and Malondialdehyde (MDA)

The method described by Heath and Packer [26] was used to measure malondialdehyde (MDA) as an index of lipid peroxidation. Briefly, 1 ml of tissue extract was added to a mixture of 2 ml trichloroacetic acid (TCA, 20%) and 1 ml thiobarbituric acid (TBA, 0.5%), heated at 95°C for 30 min, and rapidly cooled and centrifuged (Wagtech, C257-120, UK) at 4472 ×*g* for 10 min. In triplicates, 200 *µ*l aliquot of supernatant was pipetted into 96-well plates and absorbance read at both 532 nm and 600 nm, respectively, to correct for nonspecific absorbance. MDA concentration (nmol/mg protein) was calculated with its molar extinction coefficient of 1.56 × 10^−5^ M^−1^ cm^−1^:

(5) $$\begin{array}{c} \frac{\text{nmol MDA}}{\text{mg protein}}=\left[\frac{\text{absorbance }532\text{ nm}-\text{absorbance }600\text{ nm}}{\left.1.56\times 10^{5}\times \text{total protein}\right.}\right]\times 10^{6}. \end{array}$$

### 3.5. Triple Antigen-Induced Active Anaphylaxis

In this assay, the method described by Gohil et al. [27] was followed. C57BL/6 mice (25–30 g) were sensitized by single injections, respectively, of 500 *µ*l bovine serum albumin, BSA (0.5 mg ml^−1^*s.c*.), and 500 *µ*l triple antigen containing 2 × 10^7^ of *Bordetella pertussis*, *i.p*. The mice were randomized into 5 groups (*n* = 5) and given one of the following treatments daily for 10 days:

  Group I (naïve control): normal saline 10 ml kg^−1^, *p.o.*
  Group II (positive control): prednisolone 10 mg kg^−1^, *p.o.*
  Groups III–V (test groups): bergapten 3, 10, and 30 mg kg^−1^, *p.o.*, respectively

Mice were challenged with 250 *µ*l BSA (0.5 mg ml^−1^, *i*.*v*.) after 2 h of the last treatment. Mortality within 1 h after the second antigen injection was calculated using the formula:

(6) $$\begin{array}{c} \%\text{ mortality}=\left[\frac{\text{number of dead mice}}{\text{total number of mice in the group}}\right]\times 100. \end{array}$$

### 3.6. Statistical Analysis

Data were analyzed using GraphPad Prism for Windows Version 6.01 (GraphPad Prism Software, San Diego, USA). Results were presented as mean ± standard error of the mean (SEM), and the statistical differences between treatment groups compared using one-way analysis of variance (ANOVA), followed by Dunnett's post hoc test for multiple comparisons, with a 95% confidence interval. The log-rank (Mantel–Cox) test was used to analyze Kaplan–Maier survival curves. *p* < 0.05 was considered statistically significant.

## 4. Results

### 4.1. Effect of Bergapten on Compound 48/80-Induced Mast Cell Degranulation in Rat Mesenteric Tissue

**Figure 1:**
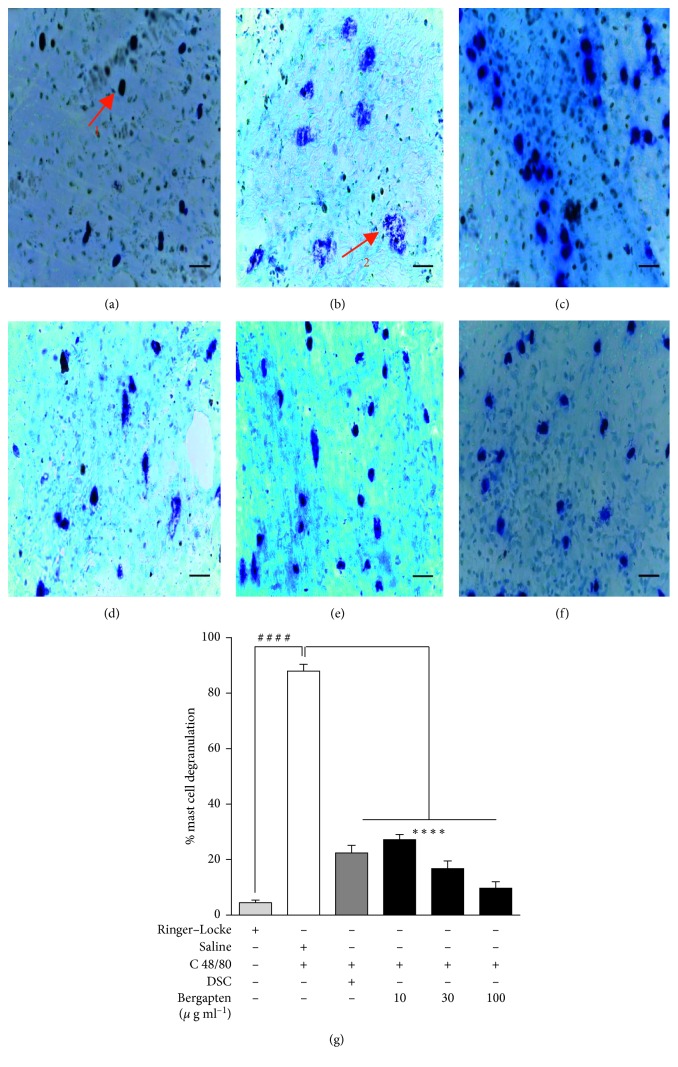
Effect of bergapten on C48/80-induced rat mesenteric mast cell degranulation. A Sprague Dawley rat was sacrificed; pieces of its mesentery excised and treated with either Ringer–Locke (a), normal saline 10 ml kg^−1^ (b), disodium cromoglycate, DSC 10 *μ*g ml^−1^ (c), or bergapten 10, 30 and 100 *μ*g ml^−1^, respectively (d–f). Tissues except the naïve control were challenged with C48/80 (1 *μ*g) for 15 min and stained with Toluidine blue (0.1%, pH 2.5) for 30 min. Representative micrographs of stained mast cells are shown (a–f) and degranulation quantified (g). Data are expressed as mean ± SEM (*n* = 5). ^*∗∗∗∗*^*p* < 0.0001 compared to the saline-treated C48/80-challenged group. ^####^*p* < 0.0001 compared to naive control group (One-way ANOVA followed by Dunnett's post hoc test). Micron bar represents 100 *μ*m.

Microscopic field observation and subsequent mast cell count of sections of rat mesenteric tissues showed purple stained intact (1) and degranulated (2) mast cells (Figures 1(a) and 1(b)). Treatment of mesenteric tissues with C48/80 resulted in significant degranulation of mast cells (Figure 1(b)) when compared to the intact mast cell histology in the Ringer–Locke naïve control group (Figure 1(a)). When administered at (10, 30, and 100 *μ*g ml^−1^), bergapten significantly inhibited mast cell degranulation (Figures 1(d)–1(f)) compared to the saline-treated C48/80-challenged mesenteric tissues (Figure 1(b)). When quantified, there was 87.98 ± 2.45% mast cell degranulation in the saline-treated C48/80 challenged mesenteric tissues compared to 4.47 ± 0.98% in the Ringer–Locke naïve control (Figure 1(g)). Treatment with bergapten at the stated doses significantly reduced percentage mast cell degranulation to 27.20 ± 1.89%, 16.73 ± 2.78%, and 9.71 ± 2.34%, respectively, when compared to the saline-treated C48/80-challenged group (Figure 1(g)), thereby stabilizing the mast cell by 60.78 ± 0.56%, 71.25 ± 0.33%, and 78.27 ± 0.11%, respectively. Disodium cromoglycate significantly reduced the extent of mast cell degranulation to 22.44 ± 2.69% compared to the saline-treated C48/80-challenged group (Figure 1(g)), giving 65.54 ± 0.24% stability to the mast cell.

### 4.2. Effect of Bergapten on Compound 48/80-Induced Systemic Anaphylaxis in Mice

**Figure 2:**
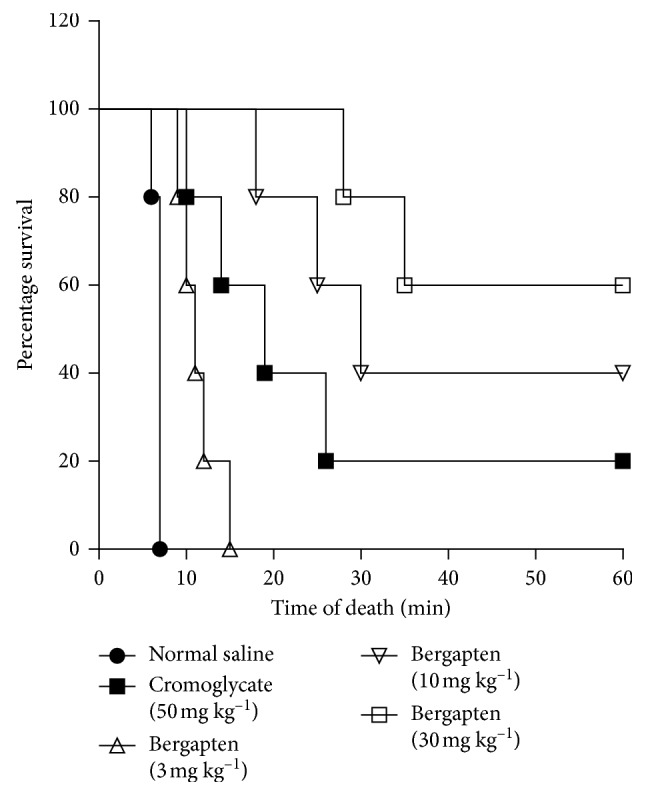
Effect of bergapten on C48/80-induced systemic anaphylaxis. C57BL/6 mice received either saline 10 ml kg^−1^, disodium cromoglycate 50 mg kg^−1^, or bergapten 3–30 mg kg^−1^, *p.o*., and challenged with C48/80 (8 mg kg^−1^, *i.p*.) 1 h later. Mortality was monitored for 1 h. Data were analyzed using the log-rank (Mantel–Cox) test. Survival curves were significant *p* < 0.0001.

Anti-anaphylactic activity of bergapten was studied using the compound 48/80-induced systemic anaphylaxis model. Intraperitoneal injection of C48/80 induced 100% fatal shock in control mice within 10 min (Figure 2). Death due to anaphylactic shock was significantly delayed in all the bergapten-treated groups when compared to the saline-treated control group. Bergapten at 3–30 mg kg^−1^ increased the latent time for death of mice compared to the saline-treated group to 15, 30, and 35 min, respectively (Figure 2). Similarly, treatment with disodium cromoglycate delayed death of mice to 26 min when compared to the saline group (Figure 2).

### 4.3. LPS-Induced Lung Inflammation in Mice

#### 4.3.1. Effect of Bergapten on Neutrophil Extravasation and Total Protein in BALF

**Figure 3:**
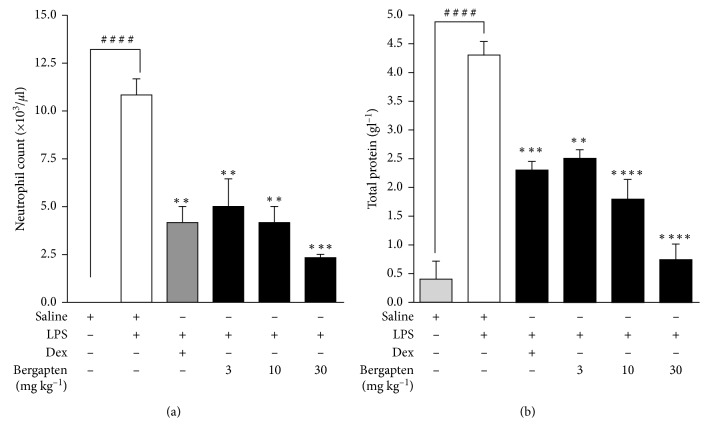
Effect of bergapten on neutrophil extravasation and total protein in BALF in LPS-induced lung inflammation. C57BL/6 mice were treated with either saline 10 ml kg^−1^, dexamethasone 10 mg kg^−1^, or bergapten 3–30 mg kg^−1^ for 1 h. Test mice were challenged with LPS and sacrificed 24 h later. BAL fluid was collected for neutrophil cell count (a) or centrifuged and supernatant used for total protein determination (b). Data are presented as cell mean count (10^3^/*µ*l) ± S.E.M (*n* = 5). ^*∗∗*^*p* < 0.01, ^*∗∗∗*^*p* < 0.001, and ^*∗∗∗∗*^*p* < 0.0001 compared to the saline-treated LPS-challenged disease control. ^####^*p* < 0.0001 compared to the naive control (one-way ANOVA followed by Dunnett's post hoc test).

Following aerosolization with LPS, there was an increased extravasation of neutrophils into the bronchoalveolar lavage fluid (BALF). Neutrophil level was significantly elevated to 10.83 ± 0.83 × 10^3^ cells/*µ*l in the saline-treated LPS-challenged disease control when compared to the saline-treated naïve control which values were below detectable levels (Figure 3(a)). Bergapten administered at 3, 10, and 30 mg kg^−1^ significantly decreased neutrophil infiltration into BALF to 5.00 ± 1.44 × 10^3^ cells/*µ*l, 4.17 ± 0.83 × 10^3^ cells/*µ*l, and 2.33 ± 0.17 × 10^3^ cells/*µ*l, respectively, when compared to the saline-treated LPS-challenged group (Figure 3(a)). Similarly, dexamethasone significantly reduced neutrophil infiltration to 4.17 ± 0.83 × 10^3^ cells/*µ*l when compared to the disease control (Figure 3(a)).

LPS-induced lung inflammation led to an increased leakage of proteins into bronchoalveolar lavage fluid. Total proteins in the BALF was significantly elevated to 4.23 ± 0.23 g l^−1^ in the saline-treated LPS-challenged disease control compared to the naive control of 0.40 ± 0.31 g l^−1^ (Figure 3(b)). All doses of bergapten 3–30 mg kg^−1^ caused significant decrease in mean protein concentration of 2.47 ± 0.15 g l^−1^, 1.77 ± 0.33 g l^−1^, and 0.73 ± 0.27 g l^−1^, respectively, compared to the saline-treated LPS-challenged control (Figure 3(b)). Dexamethasone as expected significantly reduced protein concentration to 2.27 ± 0.15 g l^−1^ (Figure 3(b)).

#### 4.3.2. Effect of Bergapten on LPS-Induced Lung Damage and Inflammatory Cell Infiltration

**Figure 4:**
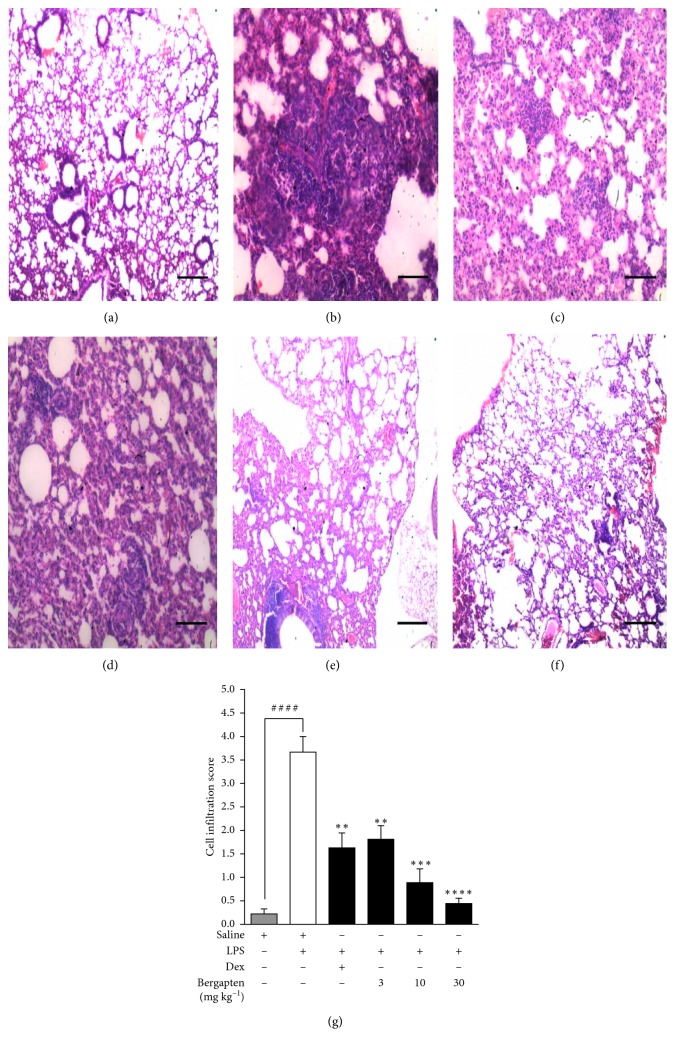
Effect of bergapten on lung damage in LPS-induced pulmonary inflammation. C57BL/6 mice were grouped and treated as naïve control, saline 10 ml kg^−1^ (a), disease control, saline 10 ml kg^−1^ (b), dexamethasone 10 mg kg^−1^ (c), or bergapten 3, 10, and 30 mg kg^−1^ (d–f) for 1 h. Test mice were challenged with LPS while naïve control received PBS only. Mice were sacrificed 24 h later. Lungs were fixed in 10% formalin and embedded in paraffin. 3 *μ*m sections were stained with H&E for histopathological examination. Degree of cell infiltration was quantified using an infiltration score described by Zare et al., (2008) with slight modifications. (g). Data are expressed as mean cell infiltration score ± SEM (*n* = 5). ^*∗∗*^*p* < 0.01, ^*∗∗∗*^*p* < 0.001, and ^*∗∗∗∗*^*p* < 0.0001 compared to saline-treated LPS-challenged disease control. ^####^*p* < 0.0001 compared to naïve control (one-way ANOVA followed by Dunnett's post hoc test). Micron bar represents 500 *μ*m.

Lung histopathological analyses were performed to evaluate the effect of bergapten on LPS-elicited lung inflammation. Normal pulmonary architecture was observed in the saline-treated naïve control group. The alveolar spaces were clear with little or no accumulation of cells around the bronchioles (Figure 4(a)). In the saline-treated LPS-challenged disease control, there was increased infiltration of inflammatory cells into the interstitium and alveoli with a distribution of cells in the peribronchial, perivascular, and also within the parenchyma area. Also, there was a distinct vascular congestion and oedema with marked decrease in alveolar air space (Figure 4(b)). Bergapten (3, 10, and 30 mg kg^−1^) attenuated these pathological changes producing mild, moderate, and marked improvement in pathological changes with less oedema and congestion and alveolar septa thickening, respectively, when compared to the saline-treated LPS-challenged disease control (Figures 4(d)–4(f)). Dexamethasone similarly attenuated the pathological changes induced by LPS (Figure 4(c)). A method described earlier by Zare et al. [23] was used to quantify these effects into a composite inflammatory score. A cell infiltration score of 0.22 ± 0.11 was recorded in the saline-treated naïve control group when compared to 3.67 ± 0.33 in the saline-treated LPS-challenged disease control (Figure 4(g)). Bergapten caused a significant reduction in cell infiltration scores to 1.81 ± 0.29, 0.89 ± 0.29, and 0.44 ± 0.11, respectively, when compared to the saline-treated LPS-challenged control at 3, 10 and 30 mg kg^−1^ (Figure 4(g)). Dexamethasone significantly reduced cell infiltration to 1.63 ± 0.32 relative to the control (Figure 4(g)).

### 4.4. Effect of Bergapten on Oxidative Stress Markers

**Figure 5:**
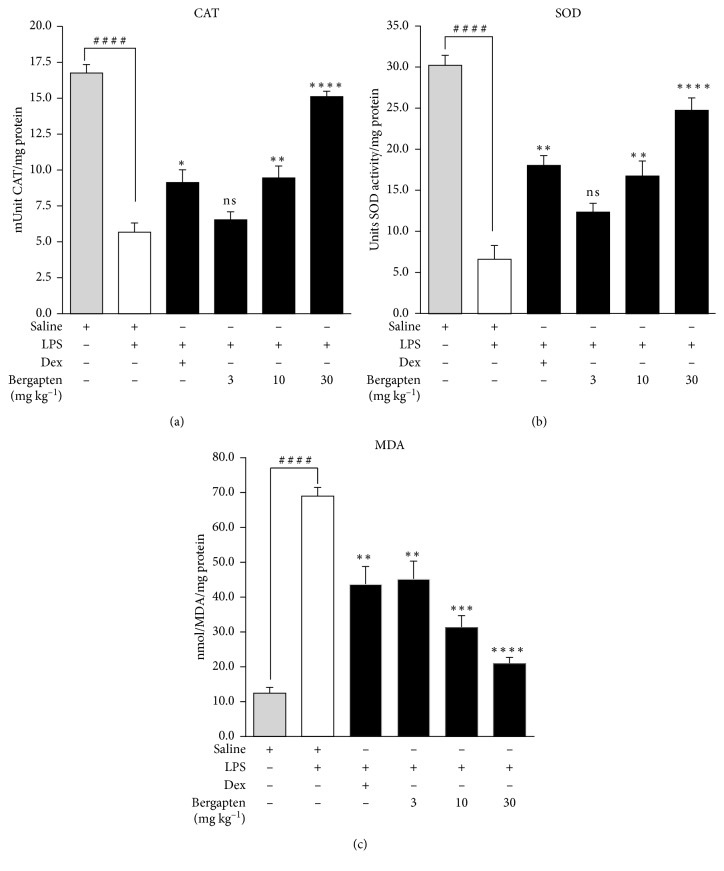
Effect of bergapten on oxidative stress markers in LPS-induced lung inflammation. C57BL/6 mice were treated with either saline 10 ml kg^−1^, dexamethasone 10 mg kg^−1^, or bergapten 3–30 mg kg^−1^ for 1 h. Test mice were challenged with LPS and sacrificed 24 h later. Lungs were harvested, processed, and supernatant analyzed quantitatively for catalase (CAT) (a), superoxide dismutase (SOD) (b), and malondialdehyde (MDA) (c). Data are expressed as mean ± SEM (*n* = 5). ^*∗*^*p* < 0.01, ^*∗∗*^*p* < 0.01, and ^*∗∗∗∗*^*p* < 0.0001 compared to saline-treated LPS-challenged disease control. ^####^*p* < 0.0001 compared to naïve control (one-way ANOVA followed by Dunnett's post hoc test). ns is not significant.

Lipopolysaccharide induced oxidative stress in the experimental animals as evidenced by the levels of the anti-oxidant enzymes measured. CAT activity in tissue supernatant was significantly reduced to 5.67 ± 0.63 mU/mg protein in the saline-treated LPS-challenged disease control compared to 16.75 ± 0.60 mU/mg protein in the saline-treated naïve control (Figure 5(a)). Bergapten administered at 10 and 30 mg kg^−1^ significantly increased CAT activity to 9.45 ± 0.83 mU/mg protein and 15.11 ± 0.37 mU/mg protein, respectively, relative to the saline-treated LPS challenged disease control. An increase to 6.54 ± 0.55 mU/mg protein with the 3 mg kg^−1^ dose of bergapten was obtained albeit insignificant (Figure 5(a)). Dexamethasone significantly elevated CAT activity to 9.13 ± 0.88 mU/mg protein relative to the saline-treated LPS challenged disease control (Figure 5(a)).

SOD activity in supernatant was reduced to 6.62 ± 1.70 U/mg protein in the saline-treated LPS challenged disease control as compared to 30.25 ± 1.23 U/mg protein in the naive control (Figure 5(b)). Administration of bergapten at 3, 10, and 30 mg kg^−1^ increased SOD activity to 12.38 ± 1.05 U/mg protein, 16.78 ± 1.83 U/mg protein, and 24.75 ± 1.52 U/mg protein, respectively, when compared to the saline-treated LPS-challenged disease control (Figure 5(b)). Dexamethasone as expected significantly increased the activity of SOD to 18.06 ± 1.21 U/mg protein compared to the LPS-challenged disease control (Figure 5(b)).

Unlike the decreased activities of CAT and SOD, there was significant increase in MDA levels to 68.79 ± 2.48 nmol/mg protein in the saline-treated LPS-challenged disease control compared to 12.38 ± 1.70 nmol/mg protein in the saline-treated naïve control (Figure 5(c)). Bergapten administered at 3, 10, and 30 mg kg^−1^ significantly decreased MDA levels to 44.79 ± 5.18 nmol/mg protein, 31.17 ± 3.42 nmol/mg protein, and 20.86 ± 1.78 nmol/mg protein, respectively, compared to the saline-treated LPS challenged disease control (Figure 5(c)). Dexamethasone significantly reduced the MDA levels to 43.48 ± 5.18 nmol/mg protein relative to that of saline-treated LPS challenged disease control (Figure 5(c)).

### 4.5. Effect of Bergapten on Triple Antigen-Induced Active Anaphylaxis

Intraperitoneal administration of bovine serum albumin (BSA) to previously sensitized and challenged mice induced anaphylactic shock and 100% mortality in the saline-treated naive control mice (Table 1). Bergapten administered at 3, 10, and 30 mg kg^−1^, respectively, protected previously sensitized and challenged mice against anaphylactic shock dose-dependently, offering 40%, 80%, and 100% survival proportions compared to the naive control group (Table 1). Similarly, prednisolone offered 80% survival in the sensitized and challenged mice when compared to the naive control group (Table 1).

## 5. Discussion

We explored the effects of bergapten (5-MOP) on allergic inflammation in two related pathways of mast cell degranulation: an IgE-independent pathway in which, respectively, compound 48/80 and lipopolysaccharide (LPS) were used to induce inflammation and an IgE-dependent pathway in which bovine serum albumin (BSA) was used as a source of allergen. The former is mediated by interaction of IgG with macrophages and basophils as the major pathways while the latter is elicited by aggregation of IgE bound to high affinity receptors (Fc*ε*RI) on surface of mast cells and basophils [28].

Mechanistically, compound 48/80 stimulates mast cells and initiates the activation of signal transduction pathways resulting in increased permeability of the lipid bilayer membrane of the mast cell, leading to perturbation. Compound 48/80 stimulates the release of phospholipase C (PLC), which eventually causes the hydrolysis of phosphatidylinositol 4, 5-biphospahte (PIP_2_), leading to the production of inositol 1,4,5-triphosphate (IP_3_) and diacylglycerol (DAG) [29]. The IP_3_ binds to receptors on the intracellular calcium ion storage sites to release calcium ions [Ca^2+^]. DAG on the other hand activates protein kinase C (PKC) to stimulate transcription factors with the release of pro-inflammatory mediators that cause allergic inflammation and anaphylactic reactions [29]. While it is established that histamine is implicated in this anaphylactic shock, it is interesting that from mast cells compound 48/80 triggers up to about 90% release of histamine relative to natural processes [30]. In the present study, the direct mast cell protection or stabilizing potential of bergapten was investigated in the compound 48/80 model of mast cell degranulation in rat mesenteric tissues. From the microscopic observation and subsequent mast cell count of sections of mesentery tissues, we could show that bergapten inhibited mast cell degranulation. Our findings suggest that bergapten possibly stabilizes the lipid bilayer membrane and/or might have inhibited histamine release by preventing compound 48/80 binding, which could have led to increased membrane permeability and perturbations. In the systemic anaphylaxis model, bergapten protected mast cells from degranulation by significantly delaying death due to anaphylactic shock.

LPS-induced inflammation is a standardized model, largely employed in the study of mechanism of activation of inflammatory pathways during a systemic inflammatory response. As expected, in our study, LPS increased protein concentration and neutrophil extravasation into BALF in the saline-treated LPS-challenged group compared to the saline control animals. LPS-induced lung inflammation causes macrophage activation with subsequent recruitment of neutrophils and leakage of proteins into the interstitium and alveoli [31]. The alveolar epithelium, interstitium, and microvascular endothelium become compromised during lung inflammation [32, 33]. It is also established that there is a mechanical enlargement of paracellular neutrophil migrating pathways, release of cytotoxic and apoptotic mediators which cause ulcerating lesions via their interactions with adjacent epithelial cells [34]. A good anti-inflammatory agent, therefore, should be able to inhibit inflammatory cell infiltration into the lung tissues. Bergapten significantly reduced both neutrophil extravasation into BALF and protein concentration. This was supported by the lung histopathological findings which showed reduced number of cellular infiltration. In the saline-only treatment group, a normal pulmonary architecture was observed compared to the LPS-challenged group with increased infiltration and distribution of cells in the peribronchial, perivascular, and within the parenchyma area. Bergapten attenuated these detrimental pathological changes and showed mild to moderate to marked improvement in lung architecture when compared to the saline-treated LPS-challenged group.

Significant correlation between LPS-induced systemic inflammation and oxidative stress has been established [35, 36]. Antioxidants are therefore needed to deal with the threat of these oxidant-induced damage [37] by diverting the enormously produced free radicals and convert them into less reactive intermediates [38]. From the study, bergapten inhibited oxidative stress in the lung tissue supernatant. Markers such as CAT and SOD in tissue supernatant were significantly reduced in the saline-treated LPS-challenged animals compared to the saline-treated animals. Antioxidant defense enzymes such as superoxide dismutase (SOD) and catalase (CAT) act on the free radicals when generated, thereby protecting cells against harmful repercussion of reactive oxygen species. Tissue levels of malondialdehyde (MDA), a product of lipid peroxidation and a positive indicator of oxidative stress [39, 40], were significantly reduced in bergapten-treated animals when compared to the saline-treated LPS-challenged animals. This apparent antioxidant effect of bergapten is consistent with some previous studies which ascribed its antioxidant activity to the presence of its phenolic content [41–44].

The involvement of IgE-dependent mast cell degranulation was also investigated in the active anaphylactic model. Active anaphylaxis induced by triple antigen alongside bovine serum albumin (BSA) is a key model to study the symptomatic effect of type I allergy. In this test, bergapten produced between 40 and 100% survival proportions against active anaphylaxis in mice previously sensitized and challenged with the antigen. The globulin fraction of BSA functions as the allergen that triggers the allergic responses. The anaphylactic reaction in mouse actively sensitized by BSA is mediated by immunoglobulin G (IgG) which mediates short latency reaction and immunoglobulin E (IgE) which mediates longer latency reaction [45, 46] and precipitating antibodies which are produced upon initial sensitization and appears in blood. Type I hypersensitivity is mediated by histamine release from mast cells. From the triple antigen-induced active anaphylaxis, bergapten might have possibly inhibited the release of histamine from the mast cells.

## 6. Conclusion

Taken together, the present study reveals the potential benefit of bergapten in managing allergy-related inflammatory conditions as demonstrated by its ability to attenuate allergic airway-induced hypersensitivity in murine models of inflammation.

## Figures and Tables

**Table 1:** Effect of bergapten on triple antigen-induced active anaphylaxis.

Treatment	No. of deaths	% mortality	Survival proportion (%)
Saline control (10 ml kg^−1^)	5	100	0.00
Prednisolone (3 mg kg^−1^)	1	20	80.00
Bergapten (mg kg^−1^)			
3.0	3	60	40.00
10.0	1	20	80.00
30.0	0	0	100.00

C57BL/6 mice were sensitized by single administration each of 500 *µ*l BSA (0.5 mg ml^−1^) and 500 *µ*l triple antigen containing 2 × 10^7^ of *Bordetella pertussis* cells. Mice were treated daily for 10 days with either saline 10 ml kg^−1^, prednisolone 10 mg kg^−1^, or bergapten 3–30 mg kg^−1^ and challenged with 250 *µ*l BSA (0.5 mg ml^−1^), *i.p*. 2 h after last treatment. Mortality was monitored for 1 h. Data were analyzed using the log-rank (Mantel–Cox) test, and survival rates were significant.

## Data Availability

Data arising from this particular study are contained within the manuscript. All data have been deposited with the Research Repository of the Kwame Nkrumah University of Science and Technology, Kumasi, Ghana. Access to these data will be considered by the authors upon request.
